# Glycoproteoforms of Osteoarthritis-associated Lubricin in Plasma and Synovial Fluid

**DOI:** 10.1016/j.mcpro.2025.100923

**Published:** 2025-02-06

**Authors:** Ali Reza Afshari, Vincent Chang, Kristina A. Thomsson, Jennifer Höglund, Elizabeth N. Browne, George Karadzhov, Keira E. Mahoney, Taryn M. Lucas, Valentina Rangel-Angarita, Henrik Ryberg, Kamlesh Gidwani, Kim Pettersson, Ola Rolfson, Lena I. Björkman, Thomas Eisler, Tannin A. Schmidt, Gregory D. Jay, Stacy A. Malaker, Niclas G. Karlsson

**Affiliations:** 1 Department of Life Sciences and Health, Faculty of Health Sciences, Oslo Metropolitan University Oslo Metropolitan University, Oslo, Norway; 2 Department of Chemistry, Yale University, New Haven, Connecticut, USA; 3 Department of Medical Biochemistry and Cell Biology, Institute of Biomedicine, Sahlgrenska Academy, University of Gothenburg, Gothenburg, Sweden; 4 Department of Laboratory Medicine, Institute of Biomedicine, University of Gothenburg, and Department of Clinical Chemistry, Sahlgrenska University Hospital, Gothenburg, Sweden; 5 Department of Life Technologies and FICAN West Cancer Centre, University of Turku, Turku, Finland; 6 Department of Orthopaedics, Institute of Clinical Sciences, The Sahlgrenska Academy, University of Gothenburg, Gothenburg, Sweden; 7 Department of Rheumatology and Inflammation Research, Institute of Medicine, Sahlgrenska Academy, University of Gothenburg, Gothenburg, Sweden; 8 Department of Clinical Sciences, Danderyd Hospital, Karolinska Institutet, Stockholm, Sweden; 9 Biomedical Engineering Department, University of Connecticut Health Centre, Farmington, Connecticut, USA; 10 Department of Emergency Medicine, Warren Alpert Medical School and Division of Biomedical Engineering, School of Engineering, Brown University, Providence, Rhode Island, USA

**Keywords:** O-linked glycosylation, biolubrication, osteoarthritis biomarker, sialylation, mucin, mucinomics, lubricin, omics, lectin, mass spectrometry, glycopeptide, synovial fluid, plasma

## Abstract

Lubricin/proteoglycan-4 (PRG-4) is a mucinous glycoprotein that lubricates cartilage and maintains normal tissue function and cell homeostasis. Altered O-glycoproteforms of lubricin have been found in osteoarthritis (OA) synovial fluid (SF), which could ostensibly be used to diagnose early onset OA. However, SF is invasive to obtain and generally would not be surveyed from otherwise healthy individuals. Thus, a plasma-based OA screening tool focused on lubricin glycosylation could be a less invasive method to aid in early-stage OA diagnosis. In this report, we used glycomics and glycoproteomics to characterize glycoproteoforms of OA lubricin in SF and plasma. We obtained near-complete sequence coverage of lubricin's mucin domain and its glycosylation using matched SF and plasma from patients with OA (N = 5). From SF lubricin we observed a spectrum of O-glycans ranging from a single GalNAcα1-Ser/Thr monosaccharide up to branched pentasaccharides. In contrast, plasma based lubricin was predominantly decorated with sialylated Galβ1-3GalNAcα1-Ser/Thr (Sialyl T). To explain the glycosylation differences observed between SF and plasma lubricin, we present splice variant-specific peptides found within the non-glycosylated region, revealing that that the longest spliceoform of lubricin was present exclusively in SF, while additional shorter splice variants could only be detected in plasma. Based on our glycoproteomic data, we developed and validated a lectin assay for lubricin, and applied this on a larger cohort of matched SF/plasma (N = 19) to confirm the glycosylation differences between SF and plasma proteoforms. Next, we leveraged our assay to screen over 100 patient with OA samples (OA patients N = 108/controls N = 38) to probe plasma lubricin as an OA biomarker. Here, we detected a decrease in α2,6 linked sialic acid in patients with OA and further show that the extent of α2,6 and α2,3 sialylation on plasma-associated lubricin correlated with patient characteristics, especially Body Mass Index (BMI).

## Highlights

• We characterized plasma and SF of lubricin glycoproteoforms from OA patients.
• 199 SF and 168 plasma O-glycosites on lubricin were detected (90% coverage).
• Plasma had sialyl T, while SF also had unsialylated T and extended structures.
• SF had the longest spliceoform of lubricin, while plasma had shorter.
• Decreased level of α2-6NeuAc as found in the OA plasma compared to controls.

## Graphical Abstract

**Figure:**
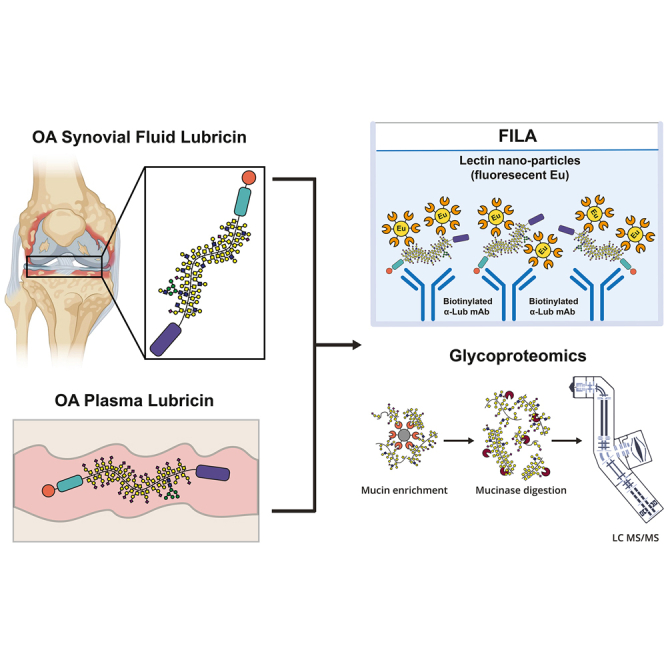

## In Brief

Lubricin from osteoarthritis patients' synovial fluid and plasma was investigated using novel tools for mucin isolation and mucinase digestion together with glycoproteomic mass spectrometry. Significant differences were observed between the two fluids in regards of lubricin glycosylation and spliceoforms. A clinical lectin assay, designed based on the observed glycosylation of lubricin, showed that osteoarthritis patients' plasma contained a decreased level lubricin α2-6 sialic acid glycovariants compared to controls.

Osteoarthritis (OA) is a degenerative joint disease wherein the protective cartilage that cushions the ends of the bones is degraded and the underlying bone is altered due to imbalances in cartilage catabolism (1). OA can affect joints such as the hands, knees, hips, and/or spine (2). The seriousness of the disease is illustrated by the fact that 10% of 60+ year old men and 18% of 60+ year old women are estimated to have symptomatic OA, with the numbers only projected to rise (3).

Currently, the diagnosis of OA is limited to radiographic evaluation, clinical examination, and detection of symptoms such as crepitus, morning stiffness of joints, joint pain, joint instability, and bone enlargement (osteophytes) (2, 4). As such, no approved molecular diagnostics for OA currently exist. In early non-chronic stages of OA, molecular diagnostics could help identify individuals who would benefit from future disease modifying osteoarthritis drugs (DMOADs) or patients to be selected for less invasive therapy compared to total joint replacement surgery (4, 5). Hence, identification of biomarker candidates, through leveraging modern -omics technologies, could aid in identifying subgroups of patients that are at risk for developing OA and that would benefit from precision medicine targeting their specific OA phenotype.

We and others have pursued the use of glycoproteoforms for the stratification of patients with OA, with a specific focus on the O-glycome of lubricin in SF (6). SF provides joint lubrication and allows for near frictionless motion. The four types of biomolecules that are involved in joint lubrication include phospholipids, hyaluronan (HA) (7), mucinous glycoproteins (*e.g.* lubricin (8)), and proteoglycans (*e.g.* aggrecan (9)). Most notably, lubricin acts as the key O-glycoprotein responsible for boundary lubrication of diarthrodial joints and for the integrity of articular cartilage (8, 10). As such, lubricin glycosylation and expression levels are altered in joint degrading diseases such as OA and rheumatoid arthritis (RA) (11). In the articular cartilage, lubricin is expressed by synoviocytes located on the synovial membrane (12) and by chondrocytes embedded in the cartilage matrix (13). Beyond the joints, lubricin is widely distributed in the body and is also expressed in the eye, liver, heart, lungs, brain, prostate, small intestine, and tendon (14, 15). Integral to lubricin's lubricating properties is its STP-rich domain (*i.e.* mucin domain) (6, 16), which is heavily glycosylated with O-linked glycans (17) that account for >50% of its total mass (18, 19). Within this domain, over 160 O-glycosites have previously been identified, including sialylated and non-sialylated T antigen O-glycans (Galβ1-3GalNAcα1-Ser/Thr) and core-2 structures such as NeuAcα2-3Galβ1-3(NeuAcα2-3Galβ1-4GlcNAcβ1-6)GalNAcα1-Ser/Thr (16, 19). In OA, an increased level of truncated O-glycans has been reported on SF lubricin, especially the Tn antigen (GalNAcα1-Ser/Thr) (20). Lubricin with truncated glycosylation acts as a signaling molecule that stimulates synoviocytes and influences the level of inflammatory cytokines, chemokines, and growth factors (20). While SF lubricin glycosylation could be valuable as a diagnostic indicator, SF is invasive to obtain and would not be collected from otherwise healthy adults. Thus, an ideal biofluid candidate for characterizing lubricin glycosylation as an early-stage OA biomarker would be less invasive, such as plasma or serum, detecting lubricin that escaped the joint capsule.

Herein, we set out to investigate the different OA glycoproteoforms of lubricin found in SF (20) and plasma using glycomic and glycoproteomic approaches. For glycoproteomics, we employed a novel workflow using mucinases to isolate and characterize lubricin (21). Previously, Malaker *et al*. used an inactive point mutant of the mucinase secreted protease of C1 esterase inhibitor (StcE^E447D^) and demonstrated that it retained its ability to bind mucins without cleaving them (22). A more recent report optimized this method by decreasing sample processing time and input, thus improving throughput for clinical samples. Thus, we reasoned that mucinases could be harnessed to both enrich lubricin from complex samples and selectively digest its heterogeneous mucin domain for glycoproteomic analysis.

Notably, the glycoproteomic experiments within would be rendered very difficult, if not impossible, without the development of electron transfer dissociation (ETD). Professor Hunt and colleagues introduced this technology 20 years ago in a seminal *PNAS* paper where they demonstrated that phosphorylation modifications remained intact on the peptide backbone (23). ETD was applied to glycoproteomics, specifically O-GlcNAcylation, by the same group several years later (24, 25, 26). Again, the soft nature of the fragmentation technique enables site-specific analysis of O-glycosites. Here, we employ ETD with and without supplemental activation (*i.e.*, EThcD) to analyze the dense O-glycosylation within the mucin domain of lubricin.

To translate the findings from glycomics and glycoproteomics, we developed, validated, and implemented a Fluorescent Immuno-Lectin Assay (FILA) to investigate clinical OA samples for differences in lubricin glycosylation between SF and plasma. Using FILA, we further demonstrated the value of plasma lubricin glycosylation for OA diagnostics. Overall, our report serves as a comprehensive study of lubricin glycosylation in OA.

## Experimental Procedures

### Ethics Declarations and Clinical Samples

Plasma samples and their controls were from a biobank maintained by Sahlgrenska University hospital containing SF and plasma from patients from two orthopedic clinics (Danderyd and Mölndal) and controls (healthy volunteers recruited for plasma donation to the Rheumatology department at Sahlgrenska University Hospital). All patients and participants had given informed consent. Ethical permission for patient screening was obtained from the ethical board in Gothenburg, Sweden (ethical application 172-15). Analysis of OA biobank samples has been approved by REK (470919) and handling of personal data by NSD (431575). The human studies reported on in the manuscript abide by the Declaration of Helsinki principles. The study was performed using samples from 183 OA patients and 41 controls. The information about the patients is summarized in Supplemental Table S2.

### Lubricin Enrichment Procedure for Glycoproteomics

Mucinases were expressed as previously described; the plasmids for His-tagged pET28a-SmEnhancin and recombinant StcE protein were kindly provided by the Bertozzi laboratory. StcE^E447D^ was conjugated to NHS-Activated Magnetic Beads (Pierce) and washed 3× with 1 ml of PBS, followed by the addition of StcE^E447D^, which was allowed to bind overnight at 4 °C. Free NHS-esters were capped by adding 100 mM Tris, pH 7.4 to the bead slurry for 20 min at 4 °C. BCA assays (Thermo-Fisher Scientific) were performed after the reactions in order to determine sufficient binding efficiency. After capping, beads were washed 3× with a high salt buffer (20 mM Tris, 500 mM NaCl) followed by a buffer without salt (20 mM Tris). OA or healthy plasma samples were brought to a final volume of 500 μl in 20 mM Tris (10 mg/ml) and EDTA was added to a final concentration of 5 mM. The StcE^E447D^-conjugated solid support (100 μl) was added to the sample and allowed to rotate at 4 °C from 5 h - overnight. After binding, the beads were washed 3× with the high salt buffer and 2× with the no salt buffer. Enriched mucins were eluted by the addition of 0.5% sodium deoxycholate in 20 mM Tris, followed by boiling the beads at 95 °C with agitation.

### Mass Spectrometry Sample Preparation

Following elution, the mucin-enriched plasma or synovial fluid was subjected to reduction, alkylation, and proteolytic digestion. To begin, dithiothreitol (Sigma) was added to a concentration of 2 mM and reacted at 65 °C for 20 min followed by alkylation in 5 mM iodoacetamide (Sigma) for 15 min in the dark at RT. Subsequently, mucinase SmE was added at an enzyme:substrate ratio of 1:20 and allowed to react overnight at 37 °C. At this point, the samples were split into two aliquots, one containing 90% and the other containing the remaining 10%. The former represented the “mucinase-only” digest that did not undergo any further proteolysis. The latter was subjected to an additional digestion with trypsin at a 1:50 enzyme:substrate ratio for 6 h at 37 °C. All reactions were quenched by adding 1 μl of formic acid (Thermo Scientific) and diluted to a volume of 200 μl prior to desalting. Addition of formic acid also caused sodium deoxycholate to precipitate out of solution, and the resulting supernatant was transferred to a new tube before desalting. Desalting was performed using 10 mg Strata-X 33 μm polymeric reversed phase SPE columns (Phenomenex). Each column was activated using 500 μl acetonitrile (ACN) (Honeywell) followed by 500 μl of 0.1% formic acid, 500 μl of 0.1% formic acid in 40% ACN, and equilibration with two additions of 500 μl of 0.1% formic acid. After equilibration, the samples were added to the column and rinsed twice with 200 μl of 0.1% formic acid. The columns were transferred to a 1.5 ml tube for elution by two additions of 150 μl of 0.1% formic acid in 40% ACN. The eluent was then dried using a vacuum concentrator (LabConco) prior to reconstitution in 10 μl of 0.1% formic acid.

### Mass Spectrometry Data Acquisition

Samples were analyzed by online nanoflow liquid chromatography-tandem mass spectrometry using an Orbitrap Eclipse Tribrid mass spectrometer (Thermo Fisher Scientific) coupled to a Dionex UltiMate 3000 HPLC (Thermo Fisher Scientific). For each analysis, 4 μl was injected onto an Acclaim PepMap 100 column packed with 2 cm of 5 μm C18 material (Thermo Fisher, 164,564) using 0.1% formic acid in water (solvent A). Peptides were then separated on a 15 cm PepMap RSLC EASY-Spray C18 column packed with 2 μm C18 material (Thermo Fisher, ES904) using a gradient from 0 to 35% solvent B (0.1% formic acid with 80% acetonitrile) in 60 min.

Full scan MS1 spectra were collected at a resolution of 60,000, an automatic gain control target of 3e5, and a mass range from *m/z* 300 to 1500. Dynamic exclusion was enabled with a repeat count of 2, repeat duration of 7 s, and exclusion duration of 7 s. Only charge states 2 to 6 were selected for fragmentation. MS2s were generated at top speed for 3 s. Higher-energy collisional dissociation (HCD) was performed on all selected precursor masses with the following parameters: isolation window of 2 m/z, 29% normalized collision energy, orbitrap detection (resolution of 7500), maximum inject time of 50 ms, and a standard automatic gain control target. An additional electron transfer dissociation (ETD) fragmentation of the same precursor was triggered if 1) the precursor mass was between m*/z* 300–1500 and 2) 3 of 8 HexNAc or NeuAc fingerprint ions (126.055, 138.055, 144.07, 168.065, 186.076, 204.086, 274.092, and 292.103) were present at *m/z* ± 0.1 and greater than 5% relative intensity. Two files were collected for each sample: the first collected an ETD scan with supplemental energy (EThcD) while the second method collected a scan without supplemental energy. Both used charge-calibrated ETD reaction times, 100 ms maximum injection time, and standard injection targets. EThcD parameters were as follows: Orbitrap detection (resolution 7500), calibrated charge-dependent ETD times, 15% nCE for HCD, maximum inject time of 150 ms, and a standard precursor injection target. For the second file, dependent scans were only triggered for precursors below *m/z* 1000, and data were collected in the ion trap using a normal scan rate.

### Mass Spectrometry Data Analysis

Raw files were searched using Byonic (version 4.5.2, Protein Metrics, Inc) against the UniProtKB/Swiss-Prot *Homo sapiens* proteome (Query: proteome:up000005640 AND reviewed:true) and a curated mucin database which was generated from a previous study (22). Briefly, the mucin database consists of nearly 350 proteins from Uniprot's annotated human proteome predicted to bear the dense O-glycosylation characteristic of mucin domains. For all samples, we used the default O-glycan database containing 9 common structures. Raw files from 5 matched OA plasma and 4 OA synovial fluid samples were first searched against the human proteome and then the curated mucin database. In both cases, files were searched with semi-specific cleavage N-terminal to Ser and Thr and six allowed missed cleavages. Samples treated with trypsin were searched with the same parameters but also allowed cleavage C-terminal to Arg or Lys. Mass tolerance was set to 10 ppm for MS1's and 20 ppm for MS2's. Met oxidation was set as a variable modification and carbamidomethyl Cys was set as a fixed modification. From the Byonic search results, glycopeptides were filtered to a score of >200 and a logprob of >2. From the remaining list of glycopeptides, the extracted ion chromatograms, full mass spectra (MS1s), and fragmentation spectra (MS2s) were investigated in XCalibur QualBrowser (Thermo) to generate a list of true-positive glycopeptides, as reported in Supplemental Data S1. Each reported glycopeptide listed in Supplemental Data S1 was manually validated from the filtered list of Byonic's reported peptides (score>200 and logprob >2) according to the following steps: The MS1 was first used to confirm the precursor mass and chosen isotope was correct. This also allowed us to identify any co-isolated species that could interfere with the MS2s and/or explain unassigned peaks. The HCD and EThcD fragmentation spectra were then investigated to identify sufficient coverage to make a sequence assignment. When possible, multiple MS2 scans were averaged to obtain a stronger spectrum. For HCD, an initial glycopeptide identification was confirmed if the presence of the precursor mass without a glycan present (*i.e.*, Y0), along with coverage of b and y ions without glycosylation. For longer peptides, we required the presence of Y0 and fragments that were expected to be abundant (*e.g.*, N-terminally to Pro, C-terminally to Asp). When the peptide contained a Pro at the C-terminus, the b_n-1_ was considered sufficient. Further, when the sequence contained oxidized Met, the Met loss from the bare mass was considered as representative of the naked peptide mass. We then used electron-based fragmentation MS2 spectra for localization. Here, all plausible localizations were considered, regardless of search result output. We confirmed the presence of fragment ions in ETD or EThcD that were between potential glycosylation sites, if sufficient c/z ions were present then a glycan mass was considered localized. For glycopeptide manual validation, extracted ion chromatograms are evaluated at the MS1 level to determine the charge and m/z of the highest abundance precursor species. Mass spectrometry data files and raw search output can be found on PRIDE with identifier PXD055049.

### MS- FILA Cross Validation of Lubricin Glycosylation

Lubricin from OA patients (n = 20) was isolated from SF samples by anion exchange chromatography as previously described (27). Lubricin-containing fractions were concentrated using 100 kD spin filters (0.5 ml Amicon Ultra, Millipore) and salt exchange was performed with 3 × 0.5 ml 0.10 M NH_4_HCO_3_, followed by speedvac to dryness. The standard mixture contained four standards: GalNAc was from Sigma-Aldrich, Galβ1-3GalNAc from Dextra (Reading, UK), and NeuAcα2-3Galβ1-3GalNAc and Sulfo Lewis^*a*^ (HSO_3_-3Galβ1-3(Fucα1-4)GlcNAc) were purchased from Carbosynth. The O-linked oligosaccharides were released as alditols by reductive β-elimination in 100 μl sodium borohydride (1.0 M) in sodium hydroxide (0.10 M) at 50 °C overnight followed by cleanup using 150 μl cation exchange media (AG50WX8, Biorad) on top of C18 SPE columns (Strata C18-E, 100 mg, Phenomenex). The oligosaccharides were dried in the speedvac, followed by repeated additions of 5 × 50 μl methanol and dried in speedvac, to evaporate borate methyl esters. Glycan standards were reduced to alditols at same conditions as above for 3 h or overnight. The oligosaccharides were dissolved in 100 μl H_2_O, followed by injection (8 μl) on a Waters UPLC-MS/MS (Acquity Xevo TQ-S) triple quadrupole mass spectrometer. Oligosaccharides were separated on porous graphitized carbon columns (100 × 2.1 mm, 3 μm particles, Hypercarb, ThermoFisher Scientific) kept at 25 °C. The gradient (36 min) consisted of 0 to 20 min 0 to 40% B (A: 10 mM ammonium bicarbonate, B: 80% acetonitrile in 10 mM ammonium bicarbonate), 20 to 23 min 40 to 100% B, 24 to 26 min wash with 1% HAc, then equilibration 26 to 36 min with 100% A. The flow rate was kept at 150 μl/min. The ESI capillary was kept on 2.5 kV. The source was at 150 °C, and the cone at 40 V. The samples were injected twice to cover a large range of glycans. The instrument was run in positive mode the first 4.35 min for analysis of GalNAcol (elution time 4 min). Positive mode transitions were the same for injections analyses, and covered the following six transitions at collision energy (CE) = 30% and with a dwell time of 0.052 ms per transition during every cycle: *m/z* 224 to 182 ([M+H]^+^-C_2_H_2_O), *m/z* 224 to 206 ([M+H]^+^-H_2_O) and also the corresponding ^13^C isotope transitions: *m/z* 225 to 183, *m/z* 225 to 207, *m/z* 246 to 182 ([M+Na]^+^-(Na+C_2_H_2_O)) and *m/z* 246 to 206 ([M+Na]^+^-(Na+H_2_O)). However, no sodium adduct transitions were detected. Transition *m/z* 224 to 182 ([M+H]^+^-C_2_H_2_O was used for quantitation. During the second period of the chromatographic run, the instrument was run in negative mode (4.35–20 min), covering nine transitions per run and at a dwell time of 0.032 ms: *m/z* 384.2 to 204.1 (CE=15); *m/z* 464.1 to 241.1 (CE=40); *m/z* 464.1 to 302.1 (CE=40); *m/z* 587.2 to 384.2 (CE=20); *m/z* 587.2 to 407.2 (CE=20); *m/z* 610.2 to 464.2 (CE=40); *m/z* 667.2 to 241.1 (CE=45); *m/z* 667.2 to 505.2 (CE=45); *m/z* 675.2 to 290.1 (CE=30); *m/z* 749.3 to 569.2 (CE=30); *m/z* 755.2 to 465.2 (CE=50); 829.2 to 667.2 (CE=50); *m/z* 878.3 to 290.1 (CE=40); *m/z* 966.3 to 290.1 (CE=50); *m/z* 1040.4 to 290.1 (CE=45); and *m/z* 1331.5 to 290.1 (CE=60) (see reference for description of transitions (28)). Collision energies were optimized using syringe infusions of saliva mucin oligosaccharides, fetuin and porcine gastric mucin oligosaccharides. A dilution series (1:2) of the standard mixture was made (0.10–90 pmol on column), with analyses of 1 to 2 standards between every three samples. Standards were analyzed using the same method as for samples, although 2 μl were injected.

### Biotinylation of Antibodies

Sulfo N-hydroxysuccinimide Biotin (sulfo-NHS-biotin) (Thermos Fisher) was used for the biotinylating of antibodies. In-house mouse IgG antibodies 1E12 (0.42 mg, for plasma-SF comparison) and 14G10 (0.71 mg, for plasma OA-Control comparison) against mucin domain of lubricin (domain mapping see Supplemental Figs. S1 and S2) was biotinylated in 2.5 ml of PBS with 20-fold molar excess of sulfo-NHS-biotin compared to antibody. The biotinylation reaction was allowed to proceed for 2 h. Excess biotin was removed using a PD-10 column (Cytiva) according to manufacturer's recommendation.

### Lectin Coating of Eu^3+^ Nanoparticles

Lectins (Vector labs) were coated covalently attached to 97 nm Fluoro-Max Dyed Carboxylate-modified Microparticles (Thermo Fisher) (20 μl). The particles were applied to 1 Nanosep 300 kDa Ω (Thermo Fisher) centrifugal filter. The Nanosep tube was centrifuged until dry at 8000 rpm for 5 minutes. Filter was washed twice with 200 μl of conjugation buffer (2-(N-morpholino)ethanesulfonic acid (MES) buffer, 50 mM, pH 6.0) and finally resuspended adding 150 μl of conjugation buffer followed by sonication with 40 pulses using an ultrasonic probe. Particles were activated by adding sulfo-NHS (230 mM dissolved in conjugation buffer, 7.4 μl) and 1-ethyl-3-(3′dimethylaminopropyl)-carbodiimide (Sigma-Aldrich, 52 mM dissolved in conjugation buffer 10.4 μl) and agitated for 15 min at room-temperature using vortex. Lectins (0.5 mg/ml dissolved in PBS, 75 μl) were added to 50 μl of particle solution and the volume was adjusted to a total of 156 μl using 500 mM MES (4.5 μl), 3.5 μl 3.0 M NaCl, 17 μl water and 6.5 μl carbonate buffer 0. 50 M, pH 9.8 and incubated and repeatedly vortexed for another 30 min. The solution was stored over night at +4 °C in rotary mixer with the exception for SNA that was just stored in +4 °C without mixing. The reaction mixture was removed on the next day using one NanoSep 300 and centrifugated (8000 rpm) for 5 min followed by washing with storage buffer twice (100 μl) (25 mM Tris, 150 mM NaCl, 0.1% NaN_3_ pH 7.8) and centrifugation at 8000 rpm for 5 min. Storage buffer (100 μl) was added on the filter and the lectin coated particles was resuspend by ultra-sonication.

### The Fluorescent Lectin-immune Assay (FILA)

Streptavidin coated micro-plates (SA96-plates, Kaivogen) were prewashed 1× with washing buffer (Kaivogen). Biotinylated mAb 1E12 and 14G10 targeting lubricin was added to each well (25 ng/well, 25 μl) and plates were incubated for 1 h in RT. After incubation the wells were washed 2× by washing buffer and plasma samples were added in triplicates. Each sample was diluted (1:150 and 1:400 or 1:5 and 1:10 for MGL) in red buffer. In addition, a serial dilution (1.3 × 10^−1^ μg/ml up to 2.5 × 10^−4^ μg/ml) of recombinant lubricin-(rhPRG4) stock conc.1300 μg/ml) were added to the plate in triplicates. Plates were then incubated for 1 h at room temperature. After the incubation, all plates were washed 2× and lectin Eu^3+^-nanoparticles (1 × 10^7^ particle/well, 25 μl). The plates were incubated for 1 h at RT and washed 6× with washing buffer. The fluorescence was read using VictorNivo multimode plate reader (PerkinElmer). The measurement operation was time-resolved fluorescence (TRF) with excitation 320 nm, and emission 615 nm wavelength. The concentration of lubricin glycoforms was calculated using MyAssays analysis software (MyAssays Ltd). The results of the FILA measurement in plasma are found in Supplemental Datas S3 and S4.

### Validation of Immune Assay

For the validation of FILA assay both spike and recovery test and test for the linearity of dilutions were performed (Validation result in Supplemental Table S1). In addition, a system-control was used for calculation of intra- and inter-assay coefficient of variations (CVs) according to recommendation (29). For spike and recovery test, diluted plasma was used adding of known amounts of rhPRG4 (spike) to selected samples. Plasma diluted was spiked with low (0.55 μg/ml), medium (1.7 μg/ml), and high (5.0 μg/ml) concentrations using rhPRG4. For recovery 1:150, 1:300, 1:600, 1:1200 diluted plasma was used and added to the plate in triplicates. In case of MGL selected plasma samples were spiked with low (0.3 μg/ml), medium (1.0 μg/ml), and high (2.9 μg/ml) concentrations using rhPRG4 and for dilution recovery 1:3, 1:6, 1:12, 1:24 diluted plasma were used. Intra-plate CV% was determined for identical samples in triplicates from the same plate. Inter-plate CV% was determined from same system-control sample in different plates.

### Experimental Design and Statistical Rationale

The analysis was performed on biobank of SF and plasma from OA patients that was due for knee joint replacement surgery. Glycoproteomic analysis was performed on a limited number of patients (N = 5) to generate glycan and protein information from plasma and SF. This information was then used to design a lectin assay for clinical screening. Synovial fluid and plasma glycosylation was compared (N = 19) and shown to reflect the glycosylation in plasma and SF shown by glycoproteomic. Plasma differences between healthy individuals and patients with OA were then screened using this assay. In total 224 samples were used for analyzing plasma lubricin glycoforms and to determine the differences in the subgroups: OA (n = 183) and controls (n = 41). Statistical analyses were performed using statistical software: SPSS (IBM) and GraphPad Prism 9 (Dotmatics). Continuous variables were summarized as mean and standard deviation or median and minimum-maximum. Assumptions such as outliers and normal distribution (Shapiro-Wilk test) were considered. All data were normalized and non-parametric Mann-Whitney U-test (unpaired) or Wilcoxon matched pairs were used to determine differences in glycoforms of lubricin between subgroups and gender (glycoform separately). The significant level was considered as *p* < 0.05 (two-tails). Correlation between BMI and age with level of FILA glycoforms was calculated using Spearman r. Significance of individual comparison (gender, age and BMI) was also subjected to multiple comparison using two-stage step-up method of Benjamini, Krieger and Yekutieli (95% confidence). Quantitative analysis of samples and standard curves were carried out regarding different types of lectins. For standard curve for MGL concentration we used linear regression and for other lectins four parameter logistic (4PL).

## Results

### Glycoproteomics Reveals Differences in Site-specific O-Glycosylation Between Plasma and SF Lubricin

Based on lubricin's dense O-glycosylation, we reasoned that mucinases could be harnessed to both enrich lubricin from complex samples and selectively digest its heterogeneous mucin domain (defined in this study as amino acids 232–1085). Previously, we developed an optimized mucin enrichment workflow which was high throughput, required low sample input (500 μg to 2.5 mg of complex sample), and compatible with in-solution mucinase digestion. By applying this optimized mucin enrichment and digestion workflow to pooled human serum, we showed that the relative intensity of mucins improved from 1.2% to 40% of the total proteome intensity, and the number of mucins identified increased by sevenfold when compared to a non-enriched control. Further, we demonstrated that we could obtain over 80% sequence coverage on lubricin and localize 168 O-glycosites, thus providing a proof-of-concept for applying this enrichment technique to OA patient samples (22).

**Fig. 1:**
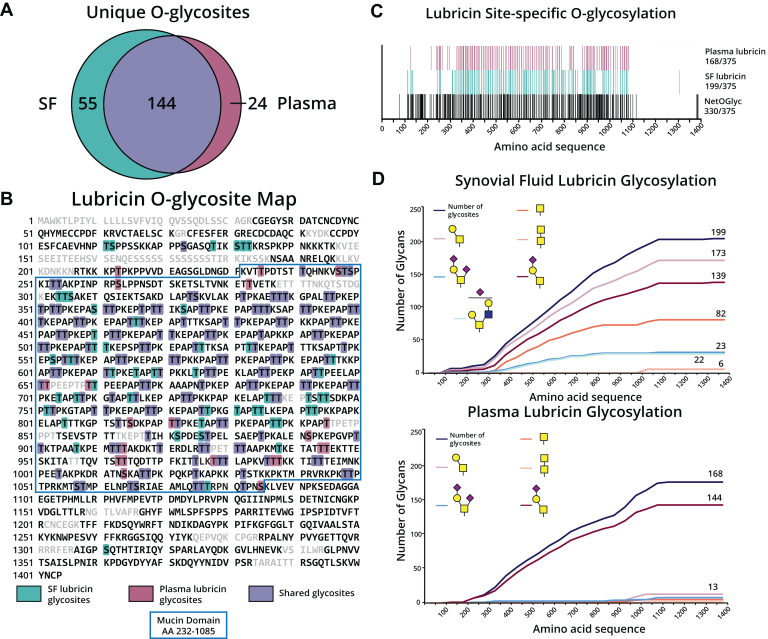
**Glycoproteomic sequencing of lubricin from mucin-enriched OA patient samples.** OA patient plasma and synovial fluid (10 mg) were subjected to StcE^E447D^ enrichment, mucinase ± trypsin digestion, and intact glycoproteomic analysis. Data was searched using Byonic against a curated mucin database and glycopeptides were manually validated. *A*, Venn diagram displaying unique O-glycosites from synovial fluid (*teal*), plasma (*magenta*) of both SF and plasma (*purple*). *B*, sequence coverage of lubricin with O-glycosites highlighted for synovial fluid (*teal*), plasma (*magenta*), of both plasma and SF (*purple*). The mucin domain of lubricin (AA 232-1085) is boxed in *blue*. *C*, graphic representation of all the identified glycans and their location in the protein sequence for synovial fluid lubricin (*top*) and plasma lubricin (*bottom*). *D*, Lubricin O-glycosites plotted against the protein sequence. Depicted are predicted O-glycosites obtained using NetOGlyc4.0 (*black*), SF (*teal*), and plasma (*magenta*) O-glycosites. Monosaccharide cartoons according to SNFG nomenclature (53).

With this method in hand, we sought to elucidate the glycosylation landscape of OA-associated lubricin (20, 27), and enriched lubricin derived from matched SF and plasma from 5 OA patients. Following MS analysis and manual glycopeptide validation, we localized a total of 199 O-glycosites on SF lubricin and 168 O-glycosites on plasma lubricin with an overall 90% sequence coverage (Fig. 1*A* and Supplemental Data S1). Among the 223 lubricin O-glycosites identified in the present study, 144 overlapped between SF and plasma, while 55 were exclusive to SF and 24 were unique to plasma lubricin (Fig. 1*A*). The unique O-glycosites from SF (teal) and plasma (magenta) lubricin along with the shared (purple) O-glycosites were mapped across the entire protein sequence (Fig. 1*B*). Most of the O-glycosites within the mucin domain were shared between SF and plasma lubricin. However, we noticed the region between amino acids 111 to 133 was predominantly occupied by SF lubricin O-glycosites, as 6 of the 7 glycosites in this region were exclusive to SF lubricin (Fig. 1, *B*, and *C*). This gave us the first indication that SF and plasma lubricin populations could differ within, or proximal to, this specific stretch of amino acids. Additionally, we compared the predicted O-glycosites from NetOGlyc 4.0 with the identified SF and plasma lubricin O-glycosites to elucidate site-specific glycosylation differences (Fig. 1*C*). NetOGlyc 4.0 uses a neural network to predict sites of *O*-linked glycosylation based on experimental evidence (30), not considering cell or tissue-specific O-GalNAc transferase expression levels. As such, this would account for the discrepancy we observed between the predicted (330 sites) and experimentally verified (223 sites) O-glycosites on OA lubricin. That being said, our method likely did not identify every possible O-glycosite on SF and plasma lubricin, given that certain glycopeptides are likely too low in abundance to be identified in a complex sample and the fact that we are still missing about 10% sequence coverage of lubricin's mucin domain. Nonetheless, we report the most comprehensive glycoproteomic map of lubricin to date with 90% sequence coverage and describe site-specific glycosylation differences between SF and plasma of patients with OA.

Next, we analyzed the glycan structures present on SF and plasma lubricin and plotted the glycans against the amino acid sequence of lubricin (Fig. 1*D*). SF lubricin primarily displayed the T antigen (52%), ST antigen (29%), and Tn antigen (12%), and to a lesser extent, core 2 structures (6%) (Fig. 1*D* and Supplemental Fig. S3). In contrast, plasma lubricin primarily exhibited the sialylated T antigen (86.5%), with minimal presence of T (6%) or Tn (2%) antigens. Overall, the glycoproteomics suggested that SF and plasma lubricin displayed distinct glycosylation patterns. To further investigate the differences in site-specific O-glycosylation between SF and plasma lubricin, we mapped O-glycan structures across a 30 amino acid stretch of the mucin domain (amino acids 324–354) as well as onto four different glycopeptide sequences (Supplemental Fig. S4, *A* and *B*). For most of the O-glycosites shown, SF lubricin displayed more microheterogeneity, supporting that the lubricin glycoforms in SF and plasma were different. Additionally, we report the first evidence of core 5/7 O-glycan structures on both SF (T1004, T1017, T1025, T1048, and T1049) and plasma (T1025) lubricin, located towards the C-terminal end of the mucin domain (Fig. 1*D*, Supplemental Fig. S5*A*, and Supplemental Data S1). Core 5 (GalNAcα1-3GalNAcα1-Ser/Thr) and core 7 (GalNAcα1-6GalNAcα1-Ser/Thr) O-glycans are isomeric and differ only in their linkages, making it difficult to distinguish them with current glycoproteomic methods. Nonetheless, core 5/7 O-glycans can be distinguished from core 3 (GlcNAcβ1-3GalNAcα1-Ser/Thr) and core 6 O-glycans (GlcNAcα1-6GalNAcα1-Ser/Thr) using the ratio of oxonium ion fragments at *m/z* 138 and *m/z* 144 in the MS^2^ spectra from HCD (31, 32, 33, 34). In Supplemental Fig. S5*B*, we show that the *m/z* 138/144 oxonium ion fragment ratio is ∼1 for the glycopeptide ATTPKPQKPTKAPK, indicating the presence of a core 5/7 O-glycan. While the role of these unique glycans on lubricin remain unclear, we add to the growing body of literature on core 5/7 *O*-glycans.

Beyond lubricin, we elucidated the broader mucinome of OA SF and plasma with this enrichment method (Supplemental Fig. S7 and Supplemental Data S2). Here, we identified 62 mucins across 4 OA SF samples and 55 mucins across 5 OA plasma samples (Supplemental Data S2). To investigate the relative abundance of lubricin with respect to the rest of the mucinome, we analyzed the intensities of matched SF and plasma from an OA patient. In OA SF, lubricin was the most abundant mucin accounting for 50% of the total intensity of all mucins, while in OA plasma, lubricin was the sixth most abundant mucin accounting for 6% of the total intensity of all mucins (Supplemental Fig. S7, *A* and *B*). Interestingly, 7 of the top 10 most abundant mucins were shared between OA SF and plasma, and gene ontology analysis of the mucins revealed molecular functions including glycosaminoglycan binding, ECM structural constituent, and ECM binding (Supplemental Fig. S5*C*).

### Plasma-Specific Lubricin Spliceoforms Differ From Synovial Fluid Lubricin

**Fig. 2:**
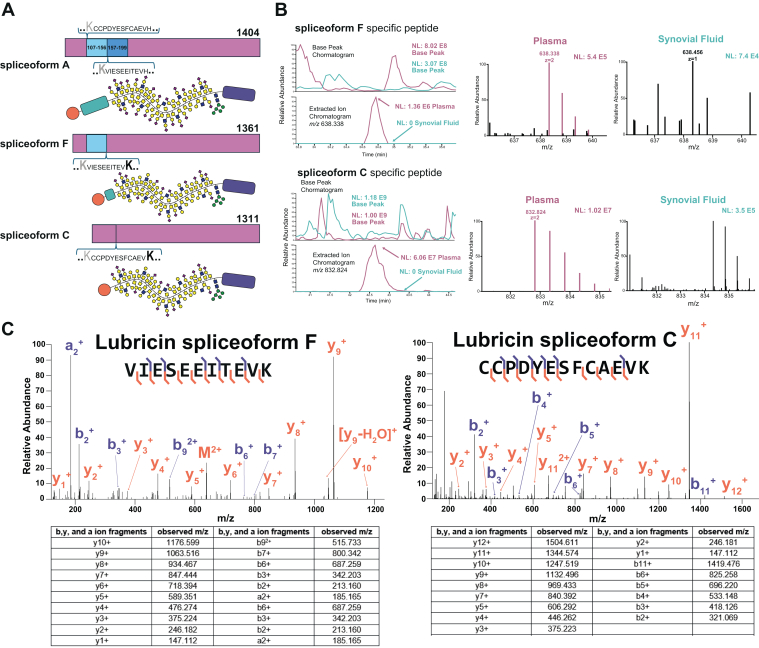
**Lubricin spliceoforms in OA patient plasma and synovial fluid.** OA patient plasma and SF after StcE^E447D^ enrichment, mucinase and trypsin digestion, followed by MS analysis and manual data interpretation. *A*, visual representation of lubricin spliceoforms and the sequence-specific tryptic peptides, which are unique to spliceoform F and C. *B*, extracted ion chromatograms and the base peak chromatogram for the spliceoform F-specific peptide (m/z 638.338, retention time 33.8–35.6 min) and the splicoform C-specific peptide (m/z 832.324, retention time 41–44.5 min). Chromatograms for plasma (*magenta*) and synovial fluid (*teal*) are overlayed. The MS1 of the monoisotopic precursor and its isotopes are shown (*right*) along with other co-isolated species. *C*, MS2 spectra for the spliceoform-specific peptides detected in plasma. Observed m/z for b^+^,y^+^, and a^+^ fragment ions are depicted on the table below.

Lubricin, encoded by the gene *PRG-4*, is reported in the UniProt database to exist as several spliceoforms. We determined which tryptic peptide sequences were specific to different lubricin spliceoforms and investigated their presence in samples of patients with OA. As shown in Figure 2*A*, the peptides VIESEEITEVK and CCPDYESFCAEVK provided evidence for spliceoforms F and C of lubricin, respectively. This is because they cannot be generated from a tryptic digest of the canonical sequence (spliceoform A) or any other distinct lubricin isoforms. After determining these sequences, we extracted the ion chromatograms (XICs) for the peptide VIESEEITEVK at the *m/z* 638.338^2+^ and CCPDYESFCAEVK at the *m/z* 832.824^2+^ for both plasma (magenta) and SF (teal) samples (Fig. 2*B*). Here, the signal intensity and precursor masses corresponding to the two lubricin spliceoforms were only present in plasma and not SF. For the two peptides detected in plasma, we confirmed the sequence assignment using MS2 (Fig. 2*C*). Overall, this indicates that plasma contain a mixture of spliceoforms A, F, and C of lubricin while SF consists only of spliceoform A of lubricin (Supplemental Fig. S6).

### Fluorescent Immuno-Lectin Assay (FILA) to Detect Glycoproteoforms of Lubricin in Plasma and SF

**Fig. 3:**
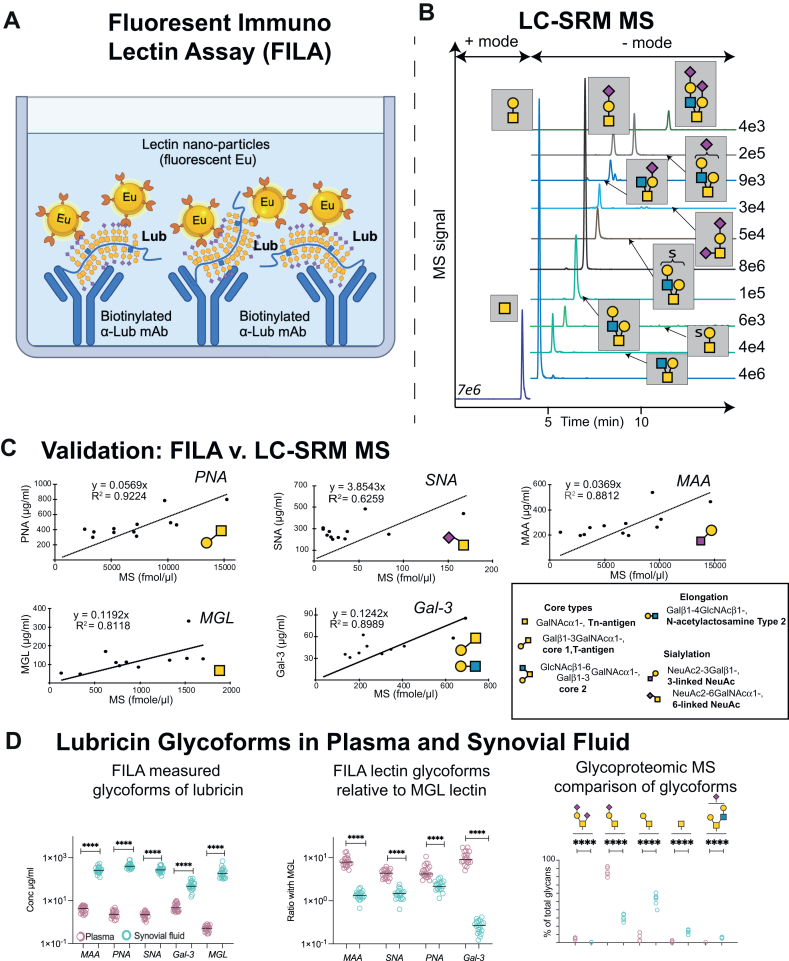
**Development of Fluorescent Immuno-Lectin Assay (FILA) to detect lubricin glycosylation differences between plasma and SF.** Schematic of Fluorescent Immuno Lectin Assay (*upper*) for measuring lubricin glycoforms sandwiched between lubricin mAb attached *via* biotin to streptavidin coated plates and Eu^3+^ fluorescent nanoparticles coated with lectins (*A*). Example of LC-SRM results quantifying glycans present on SF lubricin from an OA patient (*B*). Correlation between FILA and LC-SRM from SF lubricin evaluated using linear regression analysis (*C*). Inserted on the *right* in (*C*) is also some important cores, extensions and sialylation motifs present on lubricin. Differences in glycosylation and concentration of lubricin (*D*) Concentration of individual lubricin glycoforms using FILA comparing plasma lubricin and matched SF lubricin from late-stage knee OA patients (N = 19) (*D*, *left panel*). Ratios between individual lectin concentration and MGL concentrations to generate a concentration independent value to display major differences in glycosylation between SF and plasma lubricin (*D*, *middle panel*). Glycosylation differences between plasma and SF measured by glycoproteomics (N = 5) (*D*, *right panel*) The *p*-value has been calculated with Wilcoxon matched pairs signed rank test. ∗∗∗∗ means *p* < 0.0001. The image is partly created using BioRender.com.

To further investigate the differences between plasma and SF-derived lubricin, we developed a Fluorescent Immuno-Lectin Assay (FILA) assay (Fig. 3*A*). For this assay, we selected five lectins to cover most of the glycan epitopes found on lubricin *via* glycomic and glycoproteomic approaches. First, we used LC-coupled to a quantitative mass spectrometric (MS) Selected Reaction Monitoring (SRM) method to profile individual glycans from OA SF lubricin to benchmark the FILA (Fig. 3, *B* and *C*).

The lectins for the FILA included Macrophage Galactose-type lectin (MGL, Non-reducing terminal GalNAc, including the Tn-antigen), *Sambucus Nigra* Agglutinin (SNA, NeuAcα2-6Gal/GalNAcβ1-), *Maackia Amurensis* Agglutinin (MAA, NeuAcα2-3Galβ1-), Peanut Agglutinin (PNA, core-1/T-antigen), and Galectin-3 (Gal-3, T-antigen and Galβ1-4GlcNAc-, *N*-acetyllactosamine type 2. The FILA assay was validated using recommended tests such as spike-and-recovery, dilution-recovery, and inter-/intra-plate variation (Supplemental Table S1) (29, 35). Good agreement was observed between the FILA assays and SRM (Fig. 3*C*), with a regression coefficient for SRM/FILA usually above 0.8. The lowest regression coefficient (0.6) was found for SNA where the detected epitope did not vary enough in our sample set to reliably appreciate the correlation above the precision level of the measurement.

### Lubricin Glycoproteoforms Differ Between Plasma and SF but are Consistent Across Different Patients

We used FILA on matched plasma/SF from 19 patients with OA (Fig. 3*D*) to see how the glycosylation varied between individuals and between the two fluids. We found that the level of all glycoforms in SF was 1 to 3 orders of magnitude higher compared to plasma. For all glycan structures, the levels in plasma were below 10 μg/ml, while in SF, they were primarily detected above 100 μg/ml. These concentrations for lubricin are consistent with what have been reported in human SF and plasma previously (36, 37, 38). The highest difference in glycoform levels between SF and plasma were found for MGL, where the levels in plasma were <1 μg/ml while the levels in SF were >100 μg/ml. (Fig. 3*D*, left panel). To compare differences in lubricin glycosylation between SF (teal) and plasma (magenta), we normalized the data against the lubricin glycoforms detected by MGL and generated its ratio with the other lectins used (Fig. 3*D*, middle panel). We found that for all epitopes, the ratio was higher in plasma compared to SF, highlighting that the glycosylation of plasma-derived lubricin is markedly different when compared to SF. The data also indicated that within SF and plasma, lubricin's glycosylation was consistent across the different patients. This was confirmed by the glycoproteomic analysis when we assigned the type of glycans detected from each of the 5 plasma or SF of patients with OA (Fig. 3*D*, right panel). Having the side-by-side comparison using both glycomic FILA and glycoproteomic approaches, we can conclude that SF-derived lubricin differs from plasma *via* (a) a decreased amount of sialylated T antigen, (b) a higher level of Tn antigen, (c) more complex and heterogenous structures (*i.e.*, both core 1 and core 2 structures), and (d) an overall lower level of sialylation. Given that lubricin glycoforms differ between OA SF and plasma and are likely to have different tissue origin, the use of glycoepitopes on blood-based lubricin as a diagnostic biomarker for early-stage OA would be challenging.

### Plasma-Derived Lubricin Glycoforms in Patients With OA *Versus* Controls

**Fig. 4:**
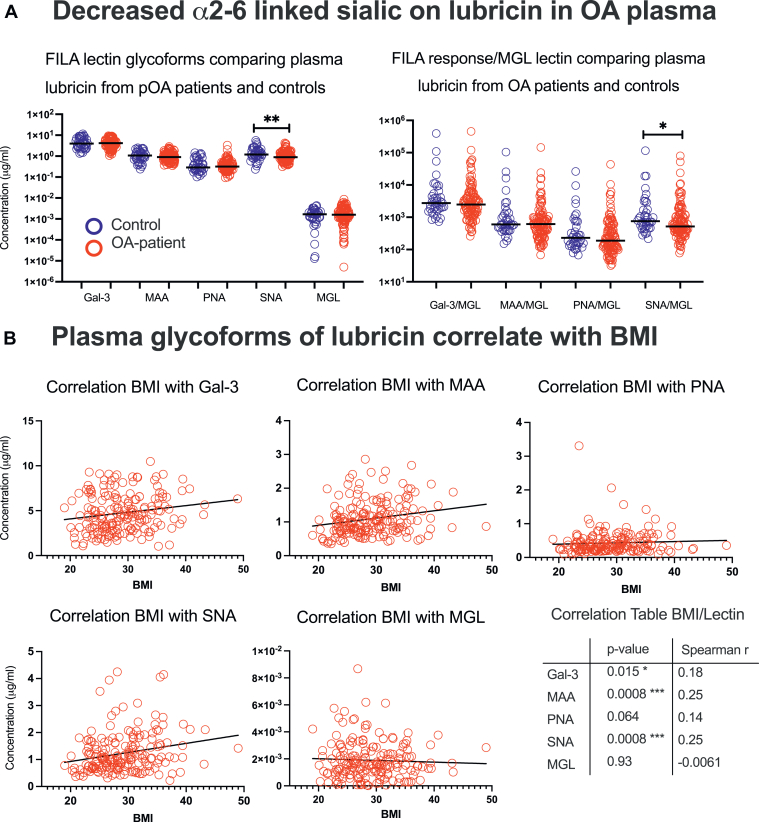
**Plasma lubricin glyco-epitopes measure by FILA.** FILA showing concentration of lubricin glycoforms measured by various lectins (*A left panel*). ∗∗ Marked for SNA (*p* = 0.0023). Ratios between individual lectin concentration and MGL concentrations to generate a concentration independent value to display major differences in glycosylation between OA and control plasma lubricin (*A right panel*). ∗ Marked for SNA/MGL (*p* = 0.0178). Non-parametric Mann-Whitney U-test (unpaired) was used to calculate the *p*-values. The data displays age, gender and BMI matched (BMI < 30) late-stage OA patients (N = 108) *versus* controls (N = 38). Correlation between plasma lubricin FILA glycoforms and BMI (*B*). The FILA results from individual lectins from plasma lubricin from late-stage knee OA ((N = 183) including both obese (BMI ≥ 30) and non-obese (BMI<30) individuals from the biobank. Inserted is also trendlines showing negative or positive correlation between individual lectins and BMI. Correlation between lectins and BMI using Spearman r and corrected for multiple comparison (95%). Unmarked difference means non-significance (*p* > 0.05).

To investigate statistical differences in glycosylation of plasma lubricin between OA and control patients, we employed our FILA method to a larger patient cohort. We selected non-obese (BMI<30) OA patients due for Total Knee Replacement (TKR) from two orthopedic clinics in Sweden (N = 108) and controls (healthy volunteers N = 38) (Supplemental Table S2, Data in Supplemental Data S3). The selection criteria for controls were individuals with no history of recurring joint problems and no history of diagnosed OA. For OA patients, we selected patients with knee OA and no history of recurring joint problems. Females were overrepresented in OA patients (59%) reflecting the increased incidence of OA in females in the population. However, no significant difference was detected in the gender, age, and BMI distribution between our OA patients and controls. Using FILA, we showed that for both OA patients and controls, the level of SNA, MAA, PNA and Gal-3 identified glycoforms of lubricin was around 10 μg/ml in plasma while the MGL-identified lubricin glycoform was 1 to 4 orders lower in magnitude (Fig. 4*A*). While the plasma lubricin levels of MAA, Gal-3, PNA, and MGL glycoforms were not significantly different between OA patients and controls (Fig. 4*A* and Supplemental Table S4), α2-6-linked sialic acid detected by SNA lectin was decreased in OA (*p* = 0.0023). This reduction also remained after removing the concentration dependency of SNA by normalizing against MGL-identified lubricin glycoform levels (*p* = 0.0178) (Fig. 4*B*). Collectively, this data suggested that OA patients displayed a decreased level of plasma lubricin glycoforms containing α2-6-linked sialic acid when compared to controls.

### Lubricin Glycoforms in Plasma Correlate With Increased BMI

We also addressed if there was a correlation between lubricin glycoforms and BMI gender and age. After selecting both obese and non-obese patients and controls from the biobank, we detected significantly positive correlations between BMI and plasma lubricin glycoforms using lectins MAA (*p* = 0.0008), SNA (*p* = 0.0008) and, to a lesser extent, Gal-3 (*p* = 0.015). No significant correlation was found for MGL and PNA (Fig. 4*C* and Supplemental Table S4, Data in Supplemental Data S4). These data indicated that an overweight BMI was associated with increased sialylation (MAA and SNA) and may have some effect on increased core1 and *N*-acetyllactosamine (Gal-3) of lubricin.

We also observed negative correlations between age and lubricin glycoforms in our OA patients using lectins MAA and SNA and a positive correlation for MGL (Supplemental Fig. S8 and Supplemental Table S5) but these differences were not as pronounced as for BMI. We also found differences in glycosylation levels between genders in the OA patients using FILA (Supplemental Table S6).

## Discussion

### Characterization of the OA Mucinome

The workflow for characterization of the OA mucinome of SF and plasma, which harnesses an inactive mucinase, enabled efficient enrichment of mucinous proteins compared to traditional and more tedious isolation procedures such as anion exchange, isopycnic centrifugation, size exclusion chromatography, and agarose or polyacrylamide electrophoresis. By circumventing these numerous enrichment steps, a mucinase-based enrichment strategy greatly reduced sample input and processing time. As a consequence, we achieved comprehensive glycoproteomic coverage of lubricin in plasma at the level of individual OA patients, overcoming previous challenges in characterizing plasma lubricin due to its low blood concentration (0.5–1.4 μg/ml). Compared to earlier studies which aimed to characterize glycosites on lubricin (16, 21, 27), we note that we attained a near 10% increase in sequence coverage and localized an additional 31 O-glycosites on SF lubricin. Further, we revealed over 60 mucin domain glycoproteins comprising the OA patient mucinome, over 50 of which have never been identified in a proteomic or glycoproteomic study of SF. Our data suggests that numerous mucins involved in OA are likely to participate in maintaining ECM homeostasis, hinting at their role in preventing cartilage degradation. Indeed, mucins such as CD248, TIMD4, and PTPRC have previously been shown to elicit an immune response and participate in wound healing and inflammation (39, 40, 41). Moving forward, it will be crucial to establish how key mucins are dysregulated at the protein level and which specific glycoepitopes change when comparing OA and healthy patient SF.

Overall, the use of mucinases for digestion continues to aid in studying mucinous molecules, where traditional proteomic proteases are often less efficient in generating O-glycopeptides in sizes that are compatible with MS based glycoproteomics. While we are now at a point where we can attain unprecedented O-glycoproteomic coverage of disease-relevant mucins from complex samples, we acknowledge there are still several limitations to our method. For instance, linkage-specific information on site-localized glycan structures is currently unattainable with our glycoproteomics workflow. A potential remedy to this challenge is the employment of Ultraviolet Photodissociation, which produces fragmentation profiles capable of elucidating both the glycan linkage and peptide backbone sequence. While this method has been applied to pure and recombinantly expressed O-glycoproteins, its utility in providing high quality data for highly complex biological samples has yet to be shown. Lastly, the presence of numerous repeat sequences within the mucin domain remains an obstacle for identification and quantitation of mucin-domain glycopeptides. This is evidenced by the fact that current glycoproteomic search algorithms continue to struggle with O-glycosite-localization and O-glycopeptide identification, thus necessitating tedious manual validation of MS1 and MS2 spectra.

### Spliceoforms of Lubricin in Plasma and SF

We observed that the presence of multiple spliceoforms which share a similar mucin domain sequence, thus complicating the analysis of resulting glycopeptides. With bottom-up glycoproteomics, it was impossible to assign shared glycopeptide sequences to a given lubricin spliceoform. An earlier study described that several lubricin spliceoforms (isoforms A to F) could result from alternative splicing of the *PRG-4* gene in different tissues, such as the cartilage and liver (15). This was accomplished by performing RT-PCR and northern blot analyses on the *PRG-4* gene across several different tissues. Interestingly, two of lubricin's splice variants were reported to result from differential splicing of the canonical isoform near the 111 to 133 amino acid stretch. However, despite transcription level evidence for the existence of these spliceoforms, little is known about their biological roles as they have never been characterized at the glycoprotein level. We found that SF lubricin was predominantly spliceoform A, while plasma contained the shorter spliceoforms C and F. Different spliceoforms in plasma compared to SF strongly suggest that that the main source of lubricin derive from different tissue origins and are likely to exhibit tissue-specific glycosylation (42). For instance, the cells which synthesize SF lubricin include synoviocytes and chondrocytes found in the joint, while plasma lubricin would predominantly derive from high-expressing tissues such as the liver (43). Therefore, use of SF lubricin circulating into the plasma as an OA diagnostic biomarker could be problematic given that SF lubricin predominantly displays T and Tn antigens. Presumably, these non-sialylated epitopes on SF lubricin escaping the joint capsule into the blood would contribute to a short biological half-life, as SF lubricin could be efficiently removed by the asialoglycoprotein receptor (ASGPR) expressed in the liver (44). As a result, it is possible that SF lubricin glycoforms entering the blood could be processed differently compared to plasma lubricin glycoforms containing higher sialylation. Looking forward, we envision that an enrichment technique such as the one in the current study could be coupled with spliceoform-specific isolation methods (*e.g.* immunoprecipitation) to provide isoform-specific analyses at the glycoproteomic level.

### FILA and Alteration of Lubricin Glycoforms in Plasma

We designed a FILA assay to evaluate most of the glycans found on lubricin (28) using 5 lectins detecting α2-3NeuAc, α2-6NeuAc, core 1/Type 2, and Tn. With glycoproteomics we also detected that core 5/7 are present on lubricin, which should be able to interact with the MGL lectin. We noticed that glycosylation of SF and plasma lubricin appear to be quite consistent between individuals. The only significant change we could detect in plasma of patients with OA *versus* controls was a decrease in SNA binding. It remains unknown whether the decrease in SNA binding to lubricin found in the OA-cohort‘s plasma (Fig. 4*A*) is caused by OA induced alterations in catabolic and/or anabolic processes. The change in SNA binding indicates that this alteration in lubricin in plasma could potentially be included in a combined assay of biomarkers for OA diagnosis/prognosis but would not suffice by itself. SNA has been described as binding to α2-6-linked sialic acid. The only α2-6-linked sialic acid detected on lubricin was directly attached to the reducing end GalNAc moiety, and we have not found that sialyl Tn-antigens are present on lubricin (28). Hence, the SNA epitope detected are likely to be more extended glycans such as NeuAcα2-6(Galβ1-3)GalNAc and NeuAcα2-6(NeuAcα2-3Galβ1-3)GalNAc. Finally, while lubricin levels in plasma have been suggested to correlate with Joint Space Narrowing (JSN) (45), this is the first time a specific glycoproteoform of lubricin in plasma has been found to be related to OA. The correlation between lubricin SNA lectin binding in plasma and BMI suggests that metabolic syndrome could be a main driver of altered O-linked glycosylation of lubricin in plasma. A potential connection between lubricin and BMI is a systemic inflammation where the inflammation is associated with being overweight (46, 47). In other causes of inflammation, such as an acute sepsis model in mice, hepatic lubricin is the most highly upregulated protein (48). This may indicate that lubricin also has an acute phase protein-like behavior. O-linked glycosylation remodeling in hepatic acute phase glycoproteins has not been widely studied, remodeling of *N*-glycosylation of acute phase proteins has been reported in both chronic and acute inflammation (49).

We also observed that plasma lubricin sialylation was negatively correlated with aging, whereas sialylation positively correlated with increased BMI. The relationship between O-linked glycosylation and age in plasma has rarely been studied, however, the sialylation of plasma *N*-linked glycosylation across different ages varies depending on protein and potentially origin (50).

## Conclusion

Using high-throughput glyco-analytics, we demonstrated that lubricin glycosylation differed significantly between OA plasma and SF, strongly suggesting that they derive from different tissues. This was further supported by the detection of different lubricin spliceoforms exclusively present in plasma and not SF. Nonetheless, our mucin-selective enrichment strategy enabled comprehensive lubricin glycoproteomic analysis from several OA patient-derived samples, thus allowing the largest targeted O-glycoproteomic characterization of a single protein across these patients. Additionally, we found that the levels of sialylated lubricin were altered in OA plasma (N = 108) compared to healthy controls (N = 38) in a large patient cohort, highlighting that plasma lubricin's O-glycosylation landscape could still be promising as an early-stage OA biomarker. More broadly, our data demonstrates that investigating plasma based O-glycome biomarkers (*e.g.* lubricin) which originate from specific diseased tissues (*e.g.* cartilage and synovium) necessitates the following considerations:

(a) Existing glycoproteoforms and splice variants of proteins arise from other healthy tissues may complicate biomarker discovery for various diseases, including OA. Isolating the different lubricin isoforms from plasma and studying their O-glycosylation landscapes separately could reveal a more diagnostically informative comparison to the canonical lubricin isoform observed in SF.
(b) Biological half-life and clearance of pathological glycovariants is important to consider, especially with regard to disease and/or cancer related Tn and T-antigens that will likely be removed by ASGPR in the liver.
(c) Disease-specific glycosylation changes of selected glycoproteins can be related to several different factors. Our data suggested that lubricin glycosylation in plasma is dependent upon OA risk factors such as BMI, age, and gender. Indeed, lubricin has been implicated in the pathogenesis of both atherosclerosis (51) and high-fat-diet induced glycose intolerance (52). Altered glycosylation of lubricin may be central to not only OA, but also other pathologies such as RA, metabolic disease, and cardiovascular diseases.

## Data Availability

Analytical data about individual patient FILA results are included in the Supplementary Material. Inquiries about additional patient meta data can be directed to the corresponding author NGK. Mass spectrometry data files and raw search output can be found on PRIDE with identifier PXD055049.

Reviewer account details:

Username: reviewer_pxd055049@ebi.ac.uk

Password: prAjONyVV4Dj.

## Supplemental data

This article contains supplemental data.

## Conflict of interests

The authors declare the following financial interests/personal relationships which may be considered as potential competing interests: NGK authored a patent involving the use of lubricin for diagnostics assigned to the company Lynxon AB, and NGK holds equity in Lynxon AB. G. J. and T. S. authored patents related to rhPRG4. G. J. and T. S. hold equity in Lubris BioPharma LLC. T. S. was also paid consultancy fees by Lubris BioPharma. SAM is an author on a Stanford patent related to the use of mucinases as research tools. The remaining authors declare no competing interests. The other authors declare that the research was conducted in the absence of any commercial or financial relationships that could be construed as a potential conflict of interest.

## Supplementary Data

Supplemental Data 1 Localization of glycosylation and glycosylation sites of lubricin from OA plasma and SF.

Supplemental Data 2 Lubricin enrichment efficiency and OA mucinome analysis.

Supplemental Data 3 and 4 Raw data from FILA for individuals BMI<30. Raw data from FILA for all individuals.
