# Supplementary material for: Glycoproteoforms of Osteoarthritis-associated Lubricin in Plasma and Synovial Fluid
Source: Mol Cell Proteomics. 2025 Feb 6;24(3):100923. doi: 10.1016/j.mcpro.2025.100923 (PMC11925169; doi:10.1016/j.mcpro.2025.100923)
Supplement: Supplementary tables [file mmc4.docx]

**Supplementary Table 1: Validation of FILA for detection of lubricin glycoforms in plasma using 5 different lectins.**

|  |  | **Spike and recovery (%)** | | | **Dilution** | **Plate CV (%)** | |
| --- | --- | --- | --- | --- | --- | --- | --- |
| **Lectins** | **Sample** | **Low (1x)** | **Medium (3x)** | **High (9x)** | **Recovery (%)** | **Intra-** | **Inter-** |
| **MAA** | 1 | 120 | 121 | 85 | 100-111 |  |  |
|  | 2 | 189 | 116 | 96 | 80-100 | 11 | 22 |
| **PNA** | 1 | 95 | 77 | 58 | 100-170 |  |  |
|  | 2 | 154 | 87 | 62 | 100-117 | 20 | 17 |
| **SNA** | 1 | 70 | 106 | 81 | 95-103 |  |  |
|  | 2 | 84 | 68 | 72 | 91-119 | 19 | 23 |
| **Gal-3** | 1 | 131 | 83 | 82 | 81-113 |  |  |
|  | 2 | 145 | 86 | 86 | 73-102 | 10 | 14 |
| **MGL** | 1 | 77 | 81 | 52 | 91-157 |  |  |
|  | 2 | 105 | 58 | 47 | 71-125 | 10 | 21 |
| **Average** |  | 117 | 88 | 72 | 88-122 | 14 | 19 |

**Supplementary Table 2: Patient Information**

| **All patients and controls** | **Control** | **OA** |
| --- | --- | --- |
| **Age (years):** median (span) | 67 (45 - 81) | 72 (48 - 93) |
| **BMI:** median (span)*^a^* | 26 (20- 29) | 30 (19 - 43) |
| **Total:** n (% of total) | 41 (18) | 183 (82) |
| **Female:** n (% within group) | 22 (54) | 108 (59) |
| **Patients and control BMI<30** |  | |
| ***^a^*Age (years):** median (span) | 67 (45 – 81) | 74 (48 – 89) |
| ***^a^*BMI:** median (span) | 26 (20 – 30) | 26 (19 – 30) |
| **Total:** n (% of total) | 38 (26) | 108 (74) |
| **Female:** n (% within group) | 19 (50) | 64 (59) |

***^a^*** Significant different in age (p = 0.005) and BMI (p = 0.009), (Control versus OA).

**Supplementary Table 3: Comparison of FILA levels between OA and control plasma lubricin glycoforms in individuals with BMI<30 and for all individuals.**

|  | ***^a^*All individuals** | | ***^b^*BMI<30** | |
| --- | --- | --- | --- | --- |
| **Glycoforms** | **Median** (Control / OA) | **p-value** | **Median** (Control / OA) | **p-value** |
| **Gal-3** | 4.195 / 4.519 | 0.3037 | 3.991 / 4.167 | 0.7441 |
| **MAA** | 1.007 / 1.010 | 0.9360 | 1.071 / 0.8970 | 0.2007 |
| **PNA** | 0.2998 / 0.3345 | 0.9912 | 0.2891 / 0.3179 | 0.6017 |
| **SNA** | 1.191 / 1.053 | 0.0610 | 1.198 / 0.8950 | 0.0023** |
| **MGL** | 0.00160 / 0.00158 | 0.4436 | 0.001681 / 0.001604 | 0.5524 |
| **Gal-3/MGL** | 2820 / 2567 | 0.6174 | 2744 / 2488 | 0.4276 |
| **MAA/MGL** | 610.0 / 635.0 | 0.7379 | 599.2 / 615.2 | 0.3703 |
| **PNA/MGL** | 240.4 / 217.1 | 0.3009 | 229.3 / 189.0 | 0.2007 |
| **SNA/MGL** | 793.2 / 618.6 | 0.0931 | 756.0 / 517.2 | 0.0178* |

***^a^* (total n=224), (Controls: n=41), (OA: n=183)**

***^b^*** **(total n=146), (Controls: n=38), (OA n=108)**

**Supplementary Table 4: Correlation between FILA glycoforms and BMI.**

| Correlation between **BMI** and lectins in control and OA samples | | | | | | |
| --- | --- | --- | --- | --- | --- | --- |
|  | | **Gal-3** | **MAA** | **PNA** | **SNA** | **MGL** |
| All (n=224) | Spearman r | 0.1653 | 0.2039 | 0.1255 | 0.1963 | -0.01051 |
|  | p-value | 0.0133* | 0.0022** | 0.0607 | 0.0032** | 0.8757 |
| **Controls** (n=41) | Spearman r | 0.1235 | 0.04906 | 0.09891 | 0.1522 | -0.06903 |
|  | p-value | 0.4418 | 0.7607 | 0.5384 | 0.3420 | 0.6681 |
| **OA** (n=183) | Spearman r | 0.1794 | 0.2453 | 0.1373 | 0.2449 | -0.00615 |
|  | p-value | 0.0151* | 0.0008*** | 0.0638 | 0.0008*** | 0.9342 |

**Supplementary Table 5: Correlation between FILA glycoforms and age.**

| Correlation between **Age** and lectins in control and OA samples | | | | | | |
| --- | --- | --- | --- | --- | --- | --- |
|  |  | **Gal-3** | **MAA** | **PNA** | **SNA** | **MGL** |
| **OA**  (n = 183) | Spearman r | -0.1084 | -0,1500 | -0.02542 | -0.1470 | 0,1769 |
|  | P-value | 0.1441 | 0.0426* | 0,7327 | 0.0471* | 0.0166* |
| **Controls**  (n = 41) | Spearman r | 0.2459 | -0.05855 | 0.2807 | 0.01311 | 0.06493 |
|  | P-value | 0.1212 | 0.7161 | 0.0755 | 0.9352 | 0.6867 |
| **All**  (n = 224) | Spearman r | -0.03545 | -0.01338 | 0.02981 | -0.0149 | 0.1683 |
|  | P-value | 0.5976 | 0.0455* | 0.6572 | 0.0258* | 0.0117* |

**Supplementary Table 6: Differences in FILA response between females and males.**

|  | ***^a^*OA** | | | | ***^b^*Control** | | | ***^c^*All** | | | |
| --- | --- | --- | --- | --- | --- | --- | --- | --- | --- | --- | --- |
| **Lectins** | Median μg/ml  (Female/Male) | | P-value | | Median μg/ml  (Female/Male) | | P-value | Median μg/ml  (Female/Male) | | P-value | |
| **Gal-3** | (5.119/3.524) |  | | <0.0001* | (3.991/4.267) |  | 0.9691 | (5.045/3.635) |  | | 0.0005* |
| **MAA** | (1.061/0.8223) |  | | 0.0105* | (0.9357/1.007) |  | 0.6885 | (1.055/0.8550) |  | | 0.0511 |
| **PNA** | (0.3643/0.2780) |  | | 0.0021* | (0.3921/0.2778) |  | 0.7860 | (0.3700/0.2800) |  | | 0.0040* |
| **SNA** | (1.147/0.8681) |  | | 0.0044* | (1.175/1.197) |  | 0.6322 | (1.155/0.9600) |  | | 0.0305* |
| **MGL** | (0.0015/0.0016) |  | | 0.9588 | (0.0013/0.0016) |  | 0.7270 | (0.0015/0.0016) |  | | 0.9199 |

The level of significance p=0.05.

***^a^***OA-patient: (Total n=183, Female n=108).

***^b^***Controls: (Total n=41, Female n=22).

***^c^***All samples: (Total n=224, Female n=130).
