# Supplementary material for: Glycoproteoforms of Osteoarthritis-associated Lubricin in Plasma and Synovial Fluid
Source: Mol Cell Proteomics. 2025 Feb 6;24(3):100923. doi: 10.1016/j.mcpro.2025.100923 (PMC11925169; doi:10.1016/j.mcpro.2025.100923)
Supplement: Supplementary Figures [file mmc5.docx]

**Supplementary Figure 1, Lubricin Antibody detection recombinant and SF lubricin using Western blot.**  Recombinant lubricin (R), synovial fluid sample (SF), bis-tris 4-20% gel and transferred to PVDF membrane. Blotted with isolated antibodies 14G10 (mucin domain of lubricin), 1E12 (mucin domain and 15C1(N-terminal) 1:10 diluted, and 2^nd^ antibody goat antimouse-HRP (1:2000).

**Supplementary Figure 2, Lubricin Antibody detection to N-terminal lubricin fragment using Western blot.** Truncated recombinant lubricin fragments labeled with GST expressed by plasmid #1 (exon 2-5, 47.8 kDa), #2 (exon 4, 34.1 kDa, #3 (exon 5. 33.1 kDa) were separated on 12.5% SDS-PAGE. After blotting to PVDF membrane and blocking, it was incubated with rabbit anti-GST antibody(1:2,000) , 14G10 (1:10), 1E12 (1:10), 15C1(1:10) and develop with HRP goat anti-moouse or goat anti-rabbit IgG (1:2000).

**Conclusion from the three antibody epitope mapping experiments (Supplementary Figure 1 and 2):** Mab 1E12 and 14G10 was shown to react with intact recombinant and SF lubricin (band >185 kDa) but not to the C-terminal fragment of 65 kDa present in human SF. Mab 15C1 interact both with intact lubricin as well as smaller fragments. Neither 1E12 nor 14G10 interacted with recombinant N-terminal fragments of lubricin, while 15C1 did. The conclusion is that both 1E12 and14G10 interact with the mucin domain, and 15C1 interacts with N-terminal.


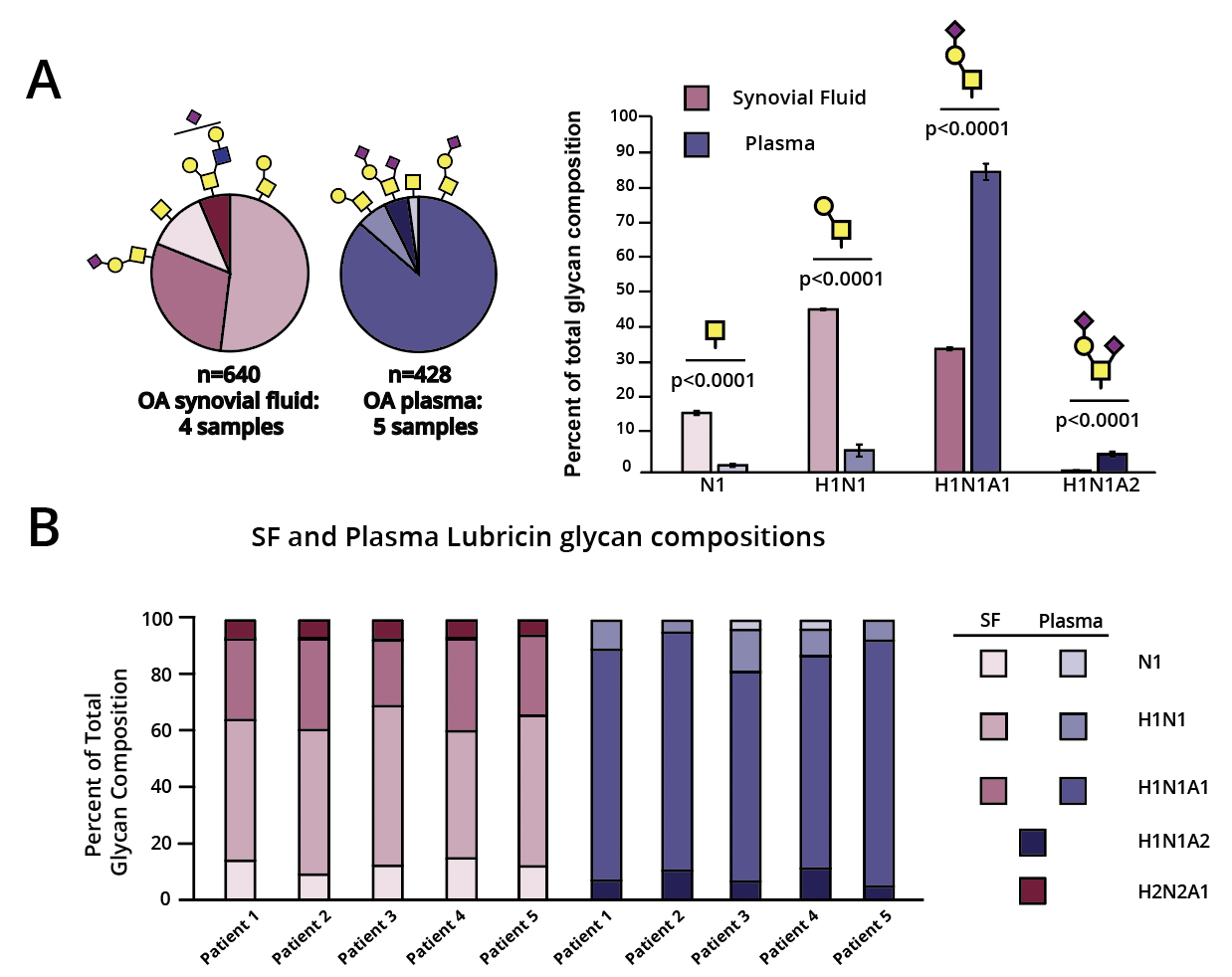


**Supplementary Figure 3. Glycan composition analysis of SF and plasma from OA patients.** (A) Glycan pie charts (left) showing the relative abundances of glycan structures in SF (red) and plasma (blue) from OA patients. Bar graph representation (right) of the relative abundances of identified core 1 O-glycan structures in SF and plasma. P-values are derived from an unpaired student’s t-test and indicated above the bars. (B) Individual glycan composition analysis for SF (red) and plasma (blue) for five OA patients. The legend is depicted on the right.


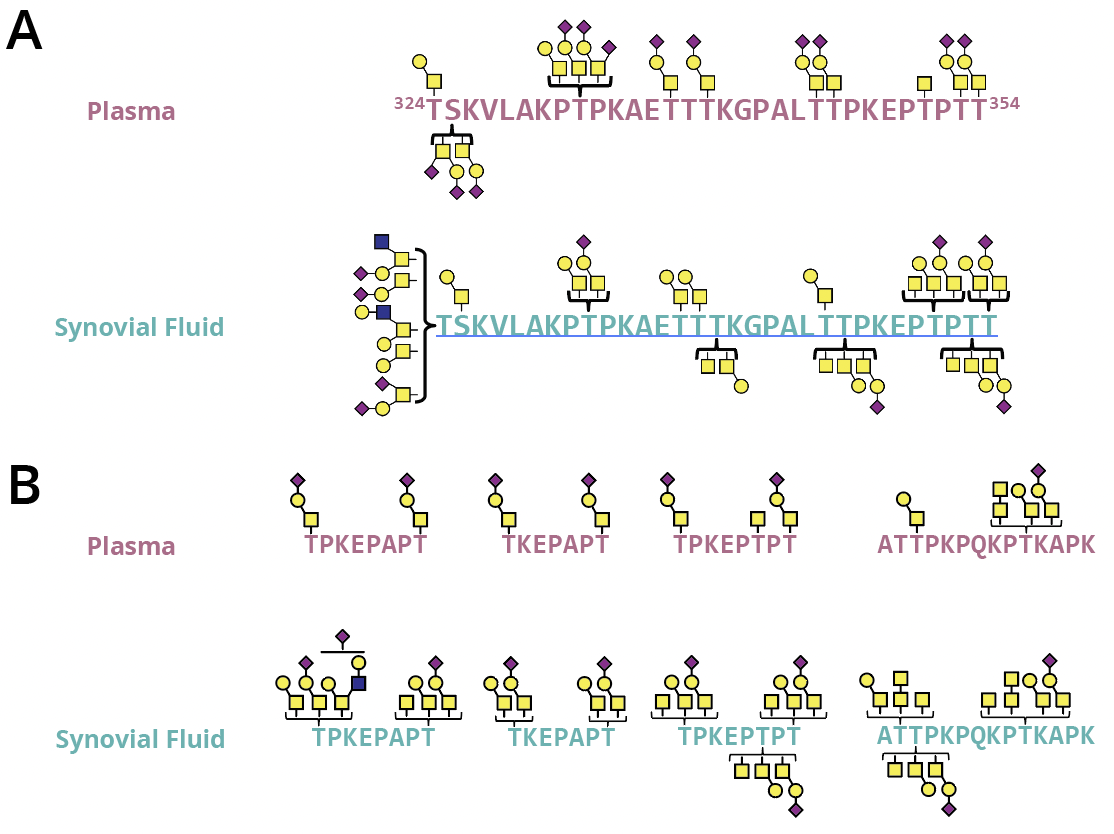


**Supplementary Figure 4. Site-specific O-glycoproteomic analysis of SF and plasma lubricin from OA patients.** Lubricin enriched from plasma and SF of OA patients was subjected to digestion with SmE mucinase and trypsin followed by MS analysis and manual data validation. (A) O-glycoproteomic map of lubricin depicting residues 324-354 from OA plasma (pink) and OA SF (teal) samples. (B) Lubricin mucin domain O-glycopeptides representing either imperfect repeats (TPKEPAPT, TKEPAPT, TPKEPTPT) or non-repeats (ATTPKPQKPTKAPK) with O-glycans localized.


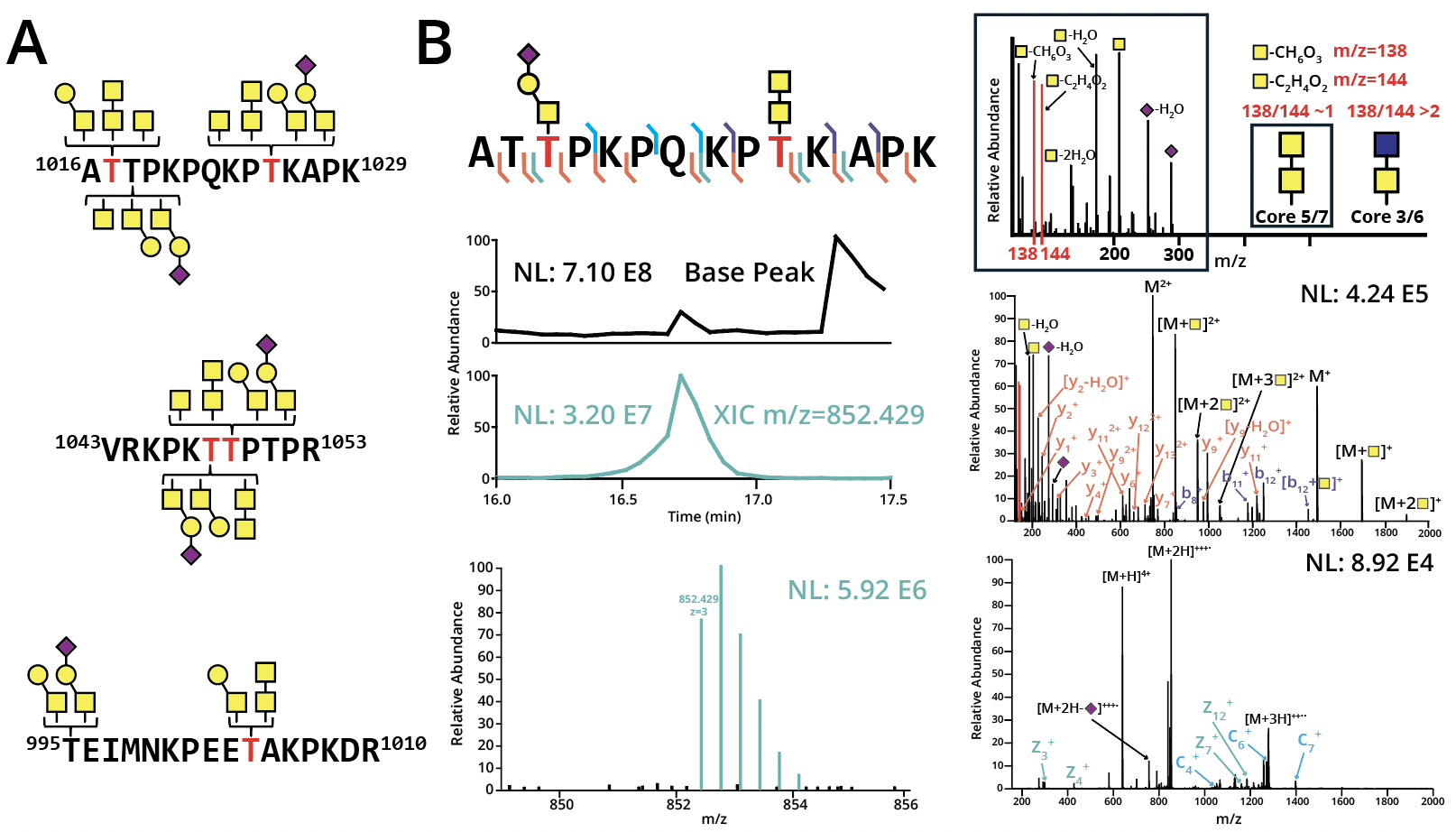


**Supplementary Figure 5. Glycoproteomic dentification of core 5/7 O-glycans** **on lubricin.** Lubricin enriched from plasma and SF of OA patients was subjected to digestion with SmE mucinase and trypsin followed by MS analysis and manual data validation. (A) Examples of identified lubricin O-glycopeptides which bear core 5/7 O-glycan structures. Threonines with core 5/7 O-glycans localized are colored in red. (B) Extracted ion chromatogram and the base peak chromatogram (left, middle) for the peptide ATTPKPQKPTKAPK (m/z 852.429, z=3, retention time 16-17.5 minutes). The MS1 of the monoisotopic precursor and its isotopes are shown (left, bottom) along with other co-isolated species. MS2 spectra are depicted on the right. The HexNAc oxonium ion fingerprint region (right, top) resulting from higher-energy collisional dissociation (HCD) is enlarged to support the core 5/7 O-glycan assignment. Briefly, the ratio of the peaks at m/z=138 and m/z=144 equaling ~1 indicates the presence of GalNAcs (i.e. core 5/7 O-glycans) and the absence of GlcNAcs (i.e. core 3/6 O-glycans). The full HCD spectrum (right, middle) shows b-ions (blue) and y-ions (orange) to support the peptide sequence assignment. The MS2 spectrum generated from electron transfer dissociation (ETD) is shown (right, bottom) to support localization of O-glycan structures.


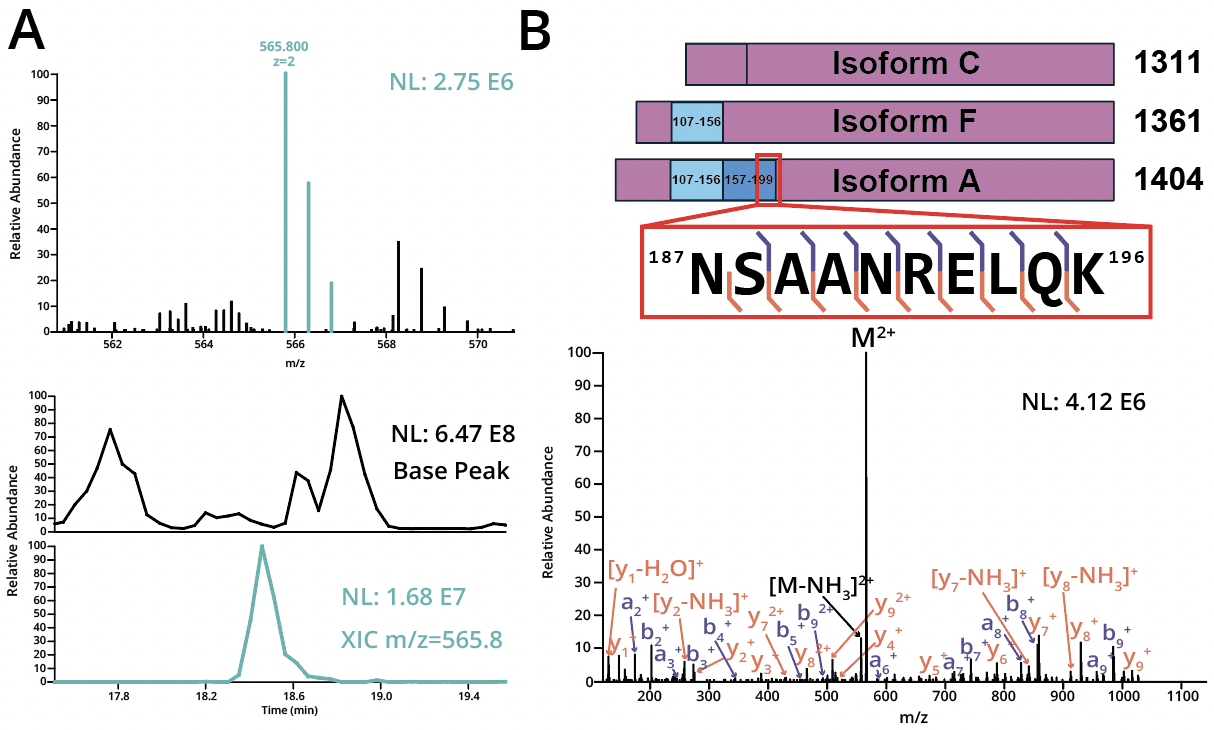


**Supplementary Figure 6. Identification of lubricin Isoform A from OA patients.** (A) Extracted ion chromatogram and the base peak chromatogram (bottom) for the isoform A-specific lubricin peptide NSAANRELQK (m/z 565.8, z=2, retention time 17.6-19.4 minutes). The MS1 of the monoisotopic precursor and its isotopes are shown (top) along with other co-isolated species. (B) The MS2 spectrum resulting from HCD shows a-ions (blue), b-ions (blue) and y-ions (orange) to support the peptide sequence assignment.

**Supplementary Figure 7: Matched SF and plasma mucinome analysis.** (A) Relative intensities of the top 10 most abundant mucins in OA SF. (B) Relative intensities of the top 10 most abundant mucins in OA plasma. (C) Gene ontology analysis of identified mucins from OA SF and plasma, displaying molecular functions.

**Supplementary Figure 8: Correlation between plasma lubricin FILA glycoforms and age.** (A-E) The FILA results from individual lectins from plasma lubricin from late-stage knee OA ((N=183) and controls (N=41). Inserted is also trendlines showing negative or positive correlation between individual lectins and Age.(F) Correlation between lectins and BMI using Spearman r.
